# FE Analysis of Critical Testing Parameters in Kolsky Bar Experiments for Elastomers at High Strain Rate

**DOI:** 10.3390/ma12233817

**Published:** 2019-11-20

**Authors:** Muhammad Salman Chaudhry, Aleksander Czekanski

**Affiliations:** Department of Mechanical Engineering, York University, 4700 Keele Street, Toronto, ON M3J 1P3, Canada; salman.chaudhry@hotmail.com

**Keywords:** kolsky bar, elastomers, high-strain rate, FEA

## Abstract

The main aim of this research is to present complete methodological guidelines for dynamic characterization of elastomers when subjected to strain rates of 100/s–10,000/s. We consider the following three aspects: (i) the design of high strain rate testing apparatus, (ii) finite element analysis for the optimization of the experimental setup, and (iii) experimental parameters and validation for the response of an elastomeric specimen. To test low impedance soft materials, design of a modified Kolsky bar is discussed. Based on this design, the testing apparatus was constructed, validated, and optimized numerically using finite element methods. Furthermore, investigations on traditional pulse shaping techniques and a new design for pulse shaper are described. The effect of specimen geometry on the homogeneous deformation has been thoroughly accounted for. Using the optimized specimen geometry and pulse shaping technique, nitrile butadiene rubber was tested at different strain rates, and the experimental findings were compared to numerical predictions.

## 1. Introduction

Low impedance materials such as elastomers are finding new applications in industries such as automotive, biomedical, aerospace, etc. A typical dynamic application of elastomers is shock absorbers for automotive and aerospace vehicles. Since hyperelastic materials like elastomers show dependency on the strain rate at which the loading is applied, researchers and engineers need to rely on properties determined under static and quasi-static conditions for such applications. Therefore, accurate models and techniques are needed to characterize elastomers in the dynamic range. In order to generate strain rate dependent constitutive models for these materials, testing at different strain rates is required.

The most common method to characterize materials at a strain rates greater than 100/s–10,000/s is the Kolsky bar technique. This apparatus has been widely used to dynamically characterize materials such as metals [1], ceramics [2], foam [3], composites [4], and smart materials [5]. The Kolsky bar is an ideal choice to characterize the stress strain response of material as a function of strain rate. A family of curves can be generated for dynamic models to accurately predict performance in impact applications. This can be done by fitting the response curves to a strain rate–dependent constitutive equation in order to derive the material parameters.

In compression, this apparatus is widely known as the Split Hopkinson pressure bar (SHPB), named after John Hopkinson (1849–1898) and his son, Bertram Hopkinson (1849–1898), known for demonstrating the transmission of stress waves in iron wires [6]. They conducted plate impact experiments to measure the pressure-time curves by a detonation or a bullet impact. Davies (1948) [7] studied this technique critically and discussed the dispersion of stress waves through long rods. In 1949, Kolsky modified the SHPB by adding an elastic bar on both sides (incident or input bar and reflected or output bar) and measured stress strain responses of materials under impact-loading conditions [8]. Kolsky also analyzed the importance of radial inertia in the specimen during deformation, which is susceptible when the strain rate is changed very rapidly [9]. The transmission of stress waves was recorded using a condenser microphone. Later, a strain-gauging technique was introduced for this purpose [10]. Krafft et al (1954) modified the Kolsky bar design to generate the loading through an accelerated projectile launch [10]. Since then, this technique has been modified and extended to incorporate tension, torsion and bend-testing of materials at high strain rates. The technique has been reviewed by Follansbee (1995) [11], Nemat-Nasser (1991) [12], Gray et al (2000) [13], and Field et al (2004) [14].

The finite element modelling of Kolsky bar has been extensively reported in previous studies, e.g., for the determination of incident pulse shaping design [15,16], modelling of pulse shaper [17], for numerical verification of the apparatus design [18], misalignment effect in the loading bars [19], and investigation on wave dispersion in bars [20]. This study aims to provide comprehensive methodology to model elastomers in a Kolsky bar experiment with a focus on specimen geometry optimization, experimental verification, and pulse shaper geometry effect.

A conventional Kolsky bar apparatus needs to be modified to test soft materials. Since soft materials are characterized by low stiffness and low elastic wave velocities, considerations such as impedance mismatch and uniform response of the specimens have to be taken into account; otherwise, it could result in a distorted and weak signal. To overcome these effects, essential modifications are essential, such as using low impedance bar materials, hollow rods [21], and pulse shapers [22].

In this paper, we examine several techniques as well as other important design parameters and requirements. A design for dynamic characterization of elastomers is presented based on the modifications needed for its use with soft materials. Investigations are made on the use of hollow transmission bar, pulse shaping technique and homogenous deformation of the specimen. Also, in order to achieve a constant strain-rate the loading pulse needs to be modified using a pulse shaper. Numerical simulations are performed using commercial explicit dynamic package (Abaqus Explicit). Various design parameters such as specimen geometry and pulse shaper have also been optimized using numerical simulations and experimental results. Finally, the optimized pulse shaper and geometry design are incorporated into the Kolsky bar for the experimental verification. A family of curves for the dynamic stress–strain responses of nitrile butadiene rubber (NBR) specimen has been generated through the modified Kolsky bar technique based on the optimized design of specimen and pulse shaper.

## 2. Kolsky Bar Design and Setup

### 2.1. Conventional Kolsky Bar

**Figure 1:**
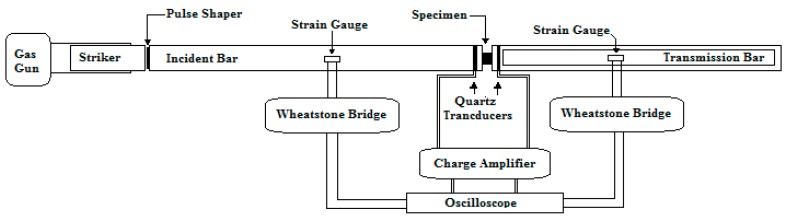
A schematic of the Kolsky setup.

A Kolsky bar experiment is the most commonly high strain rate testing equipment used to determine the dynamic response of various engineering materials. Although there is no standard design of this apparatus, most researchers use an apparatus that includes the following components (as shown in Figure 1):

- two long cylindrical elastic bars (incident, transmission);
- a striker launching mechanism (usually achieved by a gas gun);
- force/strain measuring sensors;
- bearing and base setup, for the axial alignment of bars; and
- data acquisition system.

**Figure 2:**
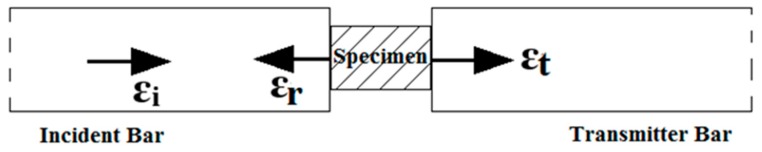
Schematic showing the incident, reflected and transmitted pulses.

In a Kolsky bar, a sample is sandwiched between the incident and transmission bar. When the striker bar impacts the incident bar, an elastic compressive stress wave is generated. This stress wave travels through the incident bar. Upon reaching the incident bar and specimen interface, part of the wave is reflected and some part of it is transmitted through the specimen into the transmission bar (Figure 2). The pulse profile for the incident, transmitted, and reflected waves are recorded as a function of time. This pulse profile is further analyzed to determine the dynamic stress strain response of the specimen [23]. A Kolsky bar setup is customized in different ways to allow for the testing of a wide variety of materials.

The traditional selection of the bar material is usually a high-strength structural material such as steel, aluminum, or nickel alloy. These high strength materials are used because the yield strength of the bar determines the maximum attainable stress. Some researchers have also used a selection of low strength materials for bars such as magnesium (45 GPa) or polymeric bars (<20 GPa) [24,25,26]. However, the disadvantage of using polymeric bars is their low heat conductivity and greater degree of dispersion and attenuation as compared to metallic bars.

In an ideal scenario, the striker bar is chosen to be the same diameter and material as of the incident and transmission bar [8]. The impacting velocity and length of the striker dictates the loading time of the pulse. This loading time is critical when testing low impedance materials. It has to be greater than the time taken for the specimen to reach stress equilibrium [8]. Under equilibrium, the amplitude of the reflected wave is directly proportional to maximum achievable strain rate. Similarly, the amplitude of the transmitted pulse is also directly proportional to the stress developed within the specimen [8,27].

### 2.2. Theory of the Kolsky Bar

The working principle of the Kolsky bar is based on the one-dimensional propagation of the compressive wave through the bars. This one-dimensional compressive elastic wave is related to the velocities that the particles experience at either interface of the bar and specimen by the following equations:

(1) $$v_{1}=C_{b}\left(\varepsilon_{i}-\varepsilon_{r}\right)$$

(2) $$v_{2}=C_{b}\left(\varepsilon_{t}\right)$$

where *ɛ_i_*, *ɛ_r_*, *ɛ_t_* are the measured incident, reflected, and transmitted strain pulses recorded from the strain gages. *C_b_* is the elastic wave speed of the bar material.

Using the above equations, the relation for averaged engineering strain integrated over the pulse duration *t* in specimen having the length *L_s_* is

(3) $$\varepsilon =\int_{0}^{t}\frac{\left(v_{1}-v_{2}\right)}{L_{s}}dt=\frac{C_{b}}{L_{s}}\int_{0}^{t}(\varepsilon_{i}-\varepsilon_{r}-\varepsilon_{t})dt$$

and the stresses at both of the interfaces of the specimen can be determined as follow:

(4) $$\sigma_{1}=\frac{A_{b}}{A_{s}}E_{b}\left(\varepsilon_{i}+\varepsilon_{r}\right)$$

(5) $$\sigma_{2}=\frac{A_{b}}{A_{s}}E_{b}\varepsilon_{t}$$

where *A_b_* and *A_s_* are the bar and specimen cross section areas and *E_b_* is the Young’s Modulus of both bars.

In order to determine the stress-strain response, we assume that the equilibrated state of stress is in the specimen (*σ*_1_ = *σ*_2_). Making this assumption we arrive at the following relation:

(6) $$\varepsilon_{r}+\varepsilon_{i}=\varepsilon_{t}$$

Thus Equations (3) and (5) can be solved to determine the stress, strain rate, and strain profile in the specimen.

(7) $$\sigma =\frac{A_{b}}{A_{s}}\cdot E_{b}\varepsilon_{t}$$

(8) $$\dot{\varepsilon }=-2\frac{C_{b}}{L_{s}}\varepsilon_{r}$$

(9) $$\varepsilon \left(t\right)=-2\frac{C_{b}}{L_{s}}\int_{0}^{t}\varepsilon_{r}dt$$

### 2.3. Kolsky Bar Design Considerations

The performance specification of the Kolsky bar apparatus is centered on the choice of specimen size and the max desired strain rate. The strain in the incident pulse can be determined by the impact velocity of the striker by the following relation:

(10) $$\varepsilon_{i}=\frac{V}{2C_{B}}$$

where *V* denotes the striker impact velocity.

A particular striking velocity is chosen to achieve the desired strain rate. The maximum amount of stress level and strain rate that can be achieved in the specimen, with a striking velocity of *V* is given by the following relations:

(11) $${\dot{\varepsilon }}_{s\left(max\right)}=\frac{V}{L_{s}}$$

(12) $$\sigma_{s}=E_{b}\left(\frac{A_{b}}{A_{s}}\right)\left(\frac{V}{2C_{b}}\right)$$

Higher levels of stress can be achieved by increasing the impact velocity or by increasing the area mismatch *A_b_*/*A_s_*. Similarly, reducing specimen length and increasing the impact velocity would increase the maximum attainable strain rate.

When selecting the bar and specimen dimensions, certain design requirements have to be met for a valid Kolsky bar. *L_b_*/*D_b_* ratio of at least 20 is required to allow the generation of one-dimensional wave propagation. Typically, this ratio is selected to be in order of 100 [28]. The diameter of the bar should be around 2–4 times the diameter of the specimen. Selecting a large ratio for *D_b_*/*D_s_* is desirable for achieving high stress but can result in punch problems. This ratio should be kept such that the bar surface does not act like a plane for the specimen. *L_s_*/*D_s_* is usually selected in between 0.6 and 1 [28] (the *L_s_*/*D_s_* ratio also depends on the type of material being tested). Soft materials have shown dependency on the length of the specimen, which could result in axial and radial inertia. The striker bar is made up of the same material and diameter as of the incident and transmitter bars to avoid wave dispersion.

#### 2.3.1. Uniform Deformation and Inertial Effects

When designing a Kolsky bar for soft materials, as discussed above, the measurements are based on the assumption of uniform deformation in the specimen. Due to the low strength, stiffness and acoustic impedance of soft materials, their mechanical response depends upon the compressive stress wave profile. Due to the low wave speed in specimen as compared to that of the bar, it takes more time for the specimen to achieve stress equilibration. The specimen can be considered to be in a state of stress equilibrium by allowing several reverberations of the stress wave inside the specimen. It requires an elastic wave to ring up almost three times inside the specimen in order to achieve near ideal stress distribution [29]. The time for a one-way trip of stress wave through the specimen can be calculated as follows:

(13) $$T=\frac{L_{s}}{C_{s}}$$

where *L_s_* is the length of the specimen and *C_s_* is the characteristic elastic wave velocity.

It can be deduced from Equation (13) that the length of the specimen is the most important variable in achieving a uniform state of stress in the specimen. Reducing the length of the specimen would help to achieve this state quickly, but on the contrary, it has been observed that a small value of *L_s_* could result in the increased frictional effect at the specimen bar interfaces, creating distortion in the recorded signal. The non-uniform deformation also results in the axial and radial inertia which is evident when testing very soft materials like elastomeric foams and gels. This induced inertia is dependent on both the loading conditions and on the specimen geometry and density. These effects cannot be ignored when optimizing the specimen dimensions.

In addition, high strain rates can also produce inertia induced stress, especially when the specimen experiences large deformation. This inertia induced stress can greatly affect the strain profiles recorded by the strain gauges [30].

#### 2.3.2. Low Transmitted Signals

Another challenge associated with testing softer materials using the Kolsky bar experiment is that the amplitude of the transmitted pulse is very low [31]. Because of the impedance mismatch between the specimen and bar materials, almost the entire the stress wave is reflected back, and only a very small portion of it is transmitted. This weak signal becomes difficult to measure by regular strain sensor and more responsive and sensitive strain gages are required. One way to address this problem is to use low impedance bars, e.g., polymer bars. Using polymer bars allows for greater wave dispersion, and spectral analysis techniques are therefore required to filter out the dispersion effect. Another approach is to use a hollow transmitter bar to help increase the amplitude of the transmitted signal up to several times [32].

#### 2.3.3. Loading Profile

Elastomers show dependency on the applied strain rate, generating different stress–strain responses for different loading rates. As a result, special care must be taken to ensure that the stress pulse profile is achieved at a constant strain rate with low rate of initial loading. A low initial rate of loading is required since acceleration induced inertia is inevitable in the initial period needed to reach a particular strain rate. A common way of avoiding this acceleration induced inertia is to use a constant rate of loading pulse with extended rise time.

#### 2.3.4. Specimen Size Effect

Specimen size needs to be properly optimized to mitigate the effects of associated radial and axial inertial responses, achieving uniform deformation and attaining a desired strain level. The specimen diameter has to be smaller than the bar diameter. Upon the assumption that the maximum diameter during deformation does not exceed the bar diameter (for the choice of desirable strain), the required diameter of the specimen can be calculated as follows:

(14) $$D_{s}=D_{b}\sqrt{1-\varepsilon }$$

For example, a bar with a diameter of 20 mm and a specimen diameter of 8 mm can achieve a maximum engineering strain close to 0.8. In addition to the selection of the specimen diameter, appropriate length of the specimen needs to be determined. This also contributes toward visible inertial effects and the stress equilibration. A thinner specimen helps in achieving a state of equilibrated stress quicker [33] but also produces visible frictional effects.

A specimen can be considered to be in equilibrated stress by comparing the stress profile on both of the faces of the specimen. Previous researchers have determined that equilibrium is reached if the ratio of the difference between the stress from either end “Δ*σ*” and the mean stress in the specimen “*σ_m_*” is less than 0.05 [34].

(15) $$R\left(t\right)=\left|\frac{\Delta \sigma }{\sigma_{m}}\right|\leq 0.05$$

Song et al. [33] demonstrated that a thin specimen maybe one of the requirements in Kolsky bar experiment but it is not sufficient enough to achieve equilibrium. Specimen design can also help in avoiding inertia in a radial direction. It was also demonstrated that this inertia reaches maximum at the center and diminishes at the edges. Therefore, using a hollow specimen could also help in addressing this challenge [35].

#### 2.3.5. Alignment of the Bars

A very important constraint in successfully conducting the Kolsky bar experiment is the alignment of the bars. It is known that misalignment could cause distortion in the recorded signal which in some cases cannot be differentiated from the actual strain signals. Because the goal is to record micro level strains, caution must be taken when setting up the apparatus. Kareem et al [19] quantified the effect of misalignment on the recorded stress strain response of the specimen. It was experimentally verified that in order to minimize this distortion, the straightness of the bars has to be at least 0.08 mm per 205 m, centerless grinded to a target diameter with a tolerance of ±0.025 mm, and finally have a perfectly perpendicular lubricated end face with a tolerance of ±0.03°.

### 2.4. Experimental Setup

For the experimental design of the Kolsky bar apparatus, the specimen geometry was optimized to be *D_s_* = 8 mm and *L_s_* = 1.6 mm. The length and the diameter were selected to be, *L_b_* = 2000 mm and *D_b_* = 20 mm respectively. These selected values satisfy the design parameter ratios that were discussed in the previous section. We get *L_b_*/*D_b_* = 100, *D_b_*/*D_s_* = 2.5, and *L_s_*/*D_s_* = 0.2. It can be noted that the *L_s_/D_s_* ratio is different when compared to the discussed range in the previous section (between 0.6 and 1). The specimen geometry was optimized carefully by taking into account the effect of L_s_ on the uniform deformation, and the equilibration of stress. Based on Equation (14) and the selection of *D_b_* and *D_s_*, we can achieve a strain level up to 0.8. The striker diameter *D_st_* is kept equal to the incident and the transmitter rods to avoid wave dispersions. The length of striker was selected to be *L_st_* = 500 mm. Relatively longer *L_st_* accounts for stress equilibration, as the loading pulse duration needs to be three times more than it takes the wave to travel back and forth inside the specimen. By using the relation $T=2L_{st}/C_{b}$, it can be seen that the choice of *L_st_* would generate a loading pulse of duration 200 µs. Therefore, it is enough for the specimen to attain uniform stress distribution and respond according to it accordingly.

From the available options of bar material, Aluminum 6061 Anodized was selected. It is evident from Equation (7) that either Young’s modulus, *E_b_* or *A_b_*/*A_s_* must be reduced in order to increase the transmitted strain. This is the main reason for selecting Aluminum as bar material in lieu of other higher strength materials that have been reportedly used in Kolsky setups. It is recommended that the striker, transmitter, and incident bar are made from the same material. Because of the impedance mismatch and very weak transmitted wave, a hollow transmitter bar was chosen to increase the amplitude of the transmitted wave. When using hollow bars, modifications need to be made to the Equation (9). Accounting, for the change in the cross-sectional area of the bars with the use of a hollow transmission bar, the following relation can be derived [32]:

(16) $$\varepsilon =\frac{C_{b}}{L}\left(1-\frac{A_{h}}{A_{b}}\right)\int_{0}^{t}\varepsilon_{i}dt-\frac{C_{b}}{L}\left(1+\frac{A_{h}}{A_{b}}\right)\int_{0}^{t}\varepsilon_{r}dt$$

To ensure uniform deformation, a pulse shaper was attached between the incident and striker bar impacting surface. Control and smoothness of the loading pulse can be achieved by using a pulse shaper as it absorbs the high frequency components of the pulse. Different choices of pulse shapers are used by researchers, such as polymer disks [32], copper disks [36] and tissue paper. In our study, pulse shaper geometry was optimized experimentally. Different dimensions of copper shaper were analyzed experimentally to determine their effect on the loading pulse profile and on homogenous deformation of elastomers.

As already discussed above, the maximum achievable strain rate depends upon the impact velocity of the striker. The most common way of accelerating the striker is by using a two staged compressed gas gun. The main supply of high-pressure gas or a compressor is used to fill the reservoir. In our testing configuration, we used a one-liter sampling cylinder with a pressure rating of up to 3000 psi. Pressure is released using a solenoid valve, with a pressure rating of 750 psi. As the velocity of the striker depends upon the initial gas pressure, choosing a high-pressure reservoir would generate a wide range of velocities in the striker. Having control over a wide range of velocities is also needed because the striking velocity relates to the strain rate achieved in the specimen. In our testing configuration, the striker could be accelerated to value up to 80 m/s upon exiting the barrel.

## 3. Numerical Simulation of Kolsky Bar

### 3.1. Finite Element Method

The finite element model to validate the design of Kolsky bar was developed in Abaqus. The high speed of loading was simulated using dynamic explicit package, capable of computing high speed transient problem with robust contact algorithm. The convergence of solution for the finite model depends on the minimum time increment associated with each computing step. A stable time increment can be perceived as the time needed for the wave to pass over the length of the smallest element present in the model. This is expressed as [37]

(17) $$\Delta t=\frac{L^{e}}{c}$$

**Figure 3:**
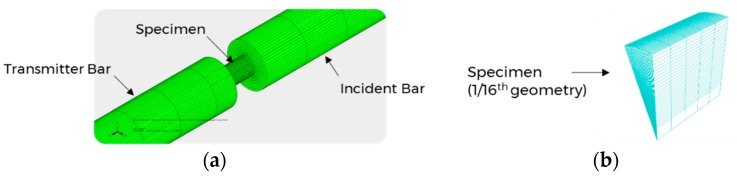
(**a**) Incident, transmitter, specimen mesh selection. and (**b**) Pulse shaper mesh.

However, reducing the time increments greatly affects the computational power required to solve the problem. Some other factors that results in increase in computational cost are (i) reducing the length of element, (ii) incorporating material with low compressibility, (ii) high stiffness of material, and (iv) reducing density of material [37]. In out finite element model, the stiffness and density of the material are fixed as these are a part of design features, which depends on the type of material being tested (in this case soft materials such as elastomers). The model of Kolsky bar was meshed with continuum elements with reduced integration (C3D8R), to reduce the computational cost. As all the parts of Kolsky bar were composed of cylindrical elements (Figure 3a), we considered a 1/16th slice of the model (Figure 3b). This also greatly reduced the computational cost of the analysis. The load carrying aluminum bar (Young Modulus 70 GPa, density 2700 kg/m^3^) were defined as elastic material with isotropic properties.

The elastomer sample is defined as hyperelastic material with Ogden order 3 strain energy potential function [38]. The model parameters are extracted from experimental data (uniaxial, planar, biaxial, and volumetric compression) through curve fitting. The calculated parameters for Treloar data [39] are summarized in Table 1. To model pulse shaper, two material models were implemented—the deviatoric response was modeled as linear elastic shear and strain rate–dependent Johnson-Cook model, while the volumetric response was modeled as Mie-Gruneisen Equation of State (E.O.S) [38]. The parameters to define these material models are summarized in Table 1.

To consider 1/16th symmetry of the cylindrical bars, the nodes on the slice surface were restricted motion in the theta direction, and only the movement in the radial direction was allowed. The end of the transmission bar was not fixed. However, this reverses the direction of the elastic wave when reflected from the free end. The impacting surface were defined as surface to surface pair, using the penalty contact algorithm. The striker is simulated by defining a velocity field over the nodes of the striker. The stress and strain values were extracted from the position where strain gauges are located in the experiment. The detailed methodology of implemented finite element model is present in reference [40] based on the design present in [41].

### 3.2. Calibration of Kolsky Bar

**Figure 4:**
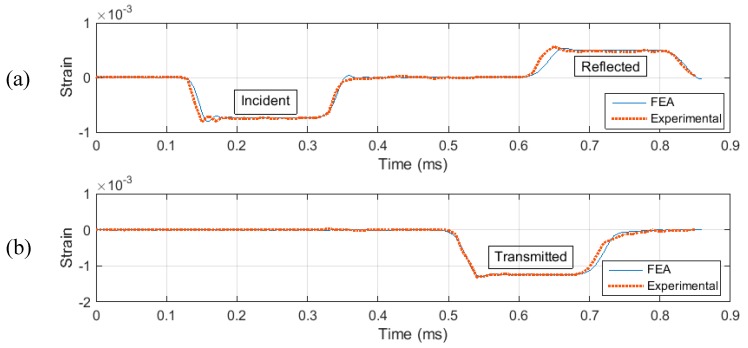
Exp. and Finite Element Model calibration, (**a**) In Incident Bar (**b**) In Transmission Bar

Before the Kolsky bar is used to test a material at dynamic loading, it requires calibration. The purpose of calibration is to ensure the accurate setup of the apparatus and alignment of the bars. The technique used to verify alignment of the bars is to launch the striker on to the incident bar, without placing the specimen in between. A good alignment between the striker and incident bar would produce a trapezoidal pulse with no distortion in the signal and an amplitude, which can be calculated analytically using Equation (10). Distortion in the signal is indicated by spikes in the baseline signal. After ensuring a good alignment between the bars, quantitative measurement can be made to calculate the elastic wave speed in the bar material. This is done by noting the time difference between the incident and reflected pulse and using the relation $C_{b}=2l_{sg}/\Delta t$, where *l_sg_* is the distance between the location of the strain gage and the specimen end of the incident bar. The time difference between the two pulses in Figure 4a was found to be 480 µs, and *l_sg_* was 1250 mm. The value of *C_b_* was calculated to be approximately 5200 m/s.

Figure 4 illustrates the experimental and FEA results when the striker is launched with a velocity of 7.5 m/s. It can be noted from Figure 4 that the amplitude of incident wave is 0.75 × 10^−4^ strain; this can be confirmed using Equation (10). The time period of the loading pulse can be calculated using the relation $T=2L_{st}/C_{b}$, which is calculated to be approximately 200 µs. Additionally, Figure 4a,b shows a very close agreement between the experimental and FEA results, meaning that the test setup is well aligned and ready to produce dynamic loadings in the test specimen.

## 4. Results

### 4.1. Specimen Geometry Optimization

When conducting Kolsky bar experiments to test elastomers, the most challenging part is facilitating homogenous deformation in the specimen. The assumption of uniform deformation is not satisfied due to the low speed of elastic waves in elastomers. As a result, a point wise deformation cannot represent the average deformation of the specimen in the thickness direction. Figure 7 represents the case of non-homogenous deformation as the compressive elastic wave travels through the specimen at different points during the loading. The specimen has the same diameter as of the bars (20 mm) and a thickness of 20 mm. At the very beginning of the loading (t = 210 µs), the elastic wave enters the specimen from the right side, and deformation only takes place in the portion that is in contact with the incident bar. As the time is increased, the deformation moves toward the transmitter/specimen interface (t = 590 µs). At no time during the loading does the specimen attains a state of averaged stress. This non uniformity of stress is attributed to axial inertia encountered in impact loadings. The axial inertia also results in radial inertia due to Poisson’s effect. This phenomenon is visible in the case of non-uniform deformation illustrated in Figure 7.

**Figure 5:**
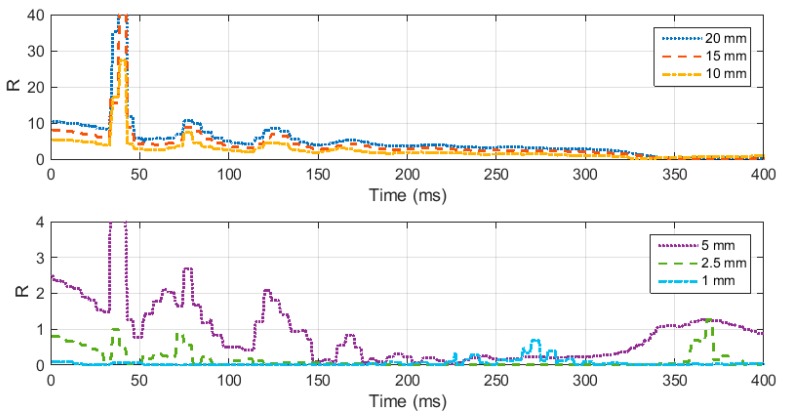
(**a**) Ratio “R” for specimen diameter 20 mm and thickness 20 mm, 15 mm, and 10 mm. (**b**) Ratio “R” for specimen diameter 20 mm and thickness 5 mm, 2.5 mm, and 1 mm.

As discussed in the previous sections, the specimen geometry needs to be optimized to ensure homogenous stress distribution in the specimen. To optimize the specimen geometry several FEA simulations were performed for test specimens with varying diameter and thickness. In order to quantify the stress distribution in the test specimen relation (15) was used to calculate the ratio *R*. Ratio *R* of less than 0.05 is sufficient to consider the specimen in an equilibrated state of stress. *R*(*t*) was calculated for specimens of various geometry and sizes. At first, the thickness was kept consistent, and various values of diameters were taken into account, ranging from 5 mm to 20 mm. Figure 5 illustrates how the ratio *R* varies throughout the loading time for the specimen with different diameter and a thickness of 1.6 mm. It can be noted that in the initial part of the loading, all the cases show non homogenous stress distribution, until the elastic wave travels several times inside the specimen and then eventually the specimens reach a state of homogenous stress. It was also inferred from the figure that the specimen with a diameter value of 5 mm showed very high value of R in the later part of the loading. It can be concluded that achieving equilibrated stress is not dependent on the diameter of the specimen, but very small values of diameter less than 8 mm can result in non-homogenous stress in later part of the loading. Therefore, when testing elastomers, a specimen diameter of more than 5 mm should be considered.

**Figure 6:**
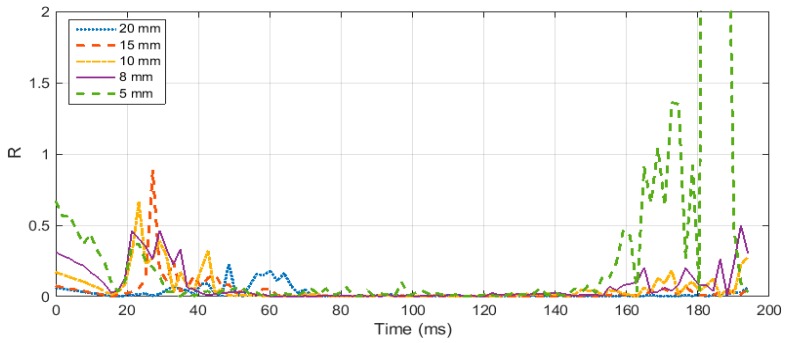
Ratio R for specimen thickness of 1.6 mm and diameter ranging from 5 mm to 20 mm.

**Figure 7:**
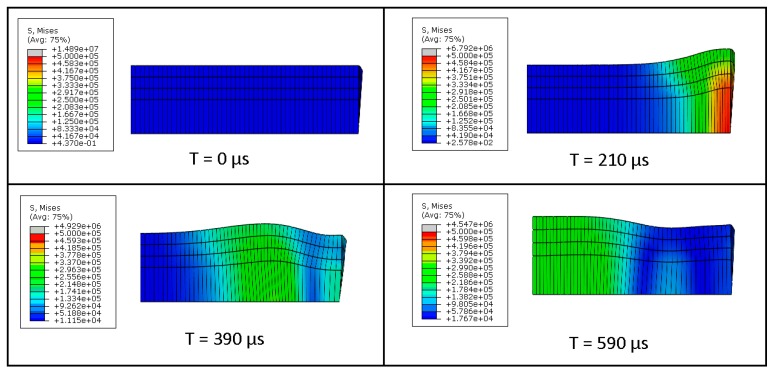
Deformation of a specimen with a thickness of 20 mm during the loading.

Next, the same ratio *R*(*t*) was calculated, taking into account a constant diameter and different values of thickness of specimen. For this case different thicknesses ranging from 1 mm to 20 mm were considered. The results are illustrated in Figure 6. It can be noted from Figure 6 that all the specimen with thickness greater than 5 mm stays in a state of non-homogenous state of stress throughout the loading time. Figure 7 shows the deformation of a 20 mm thickness specimen during the loading. It can be clearly seen from the stress distribution at different time points during the loading that the specimen fails to achieve uniform state of stress. This non-uniform state of stress is attributed to inertial and radial inertia produced. Only the specimens with thicknesses equal to or less than 5 mm achieved a state of ideal loading. As the thickness of the specimen is decreased, the time to equilibrium is decreased and the specimen becomes closer to stress equilibrium with a reduced specimen. However, a very thin specimen may result in interfacial frictional effects. Based on the results collected from this study, it is recommended to use a specimen thickness of less than 2.5 mm.

### 4.2. Pulse Shaper Analysis

**Figure 8:**
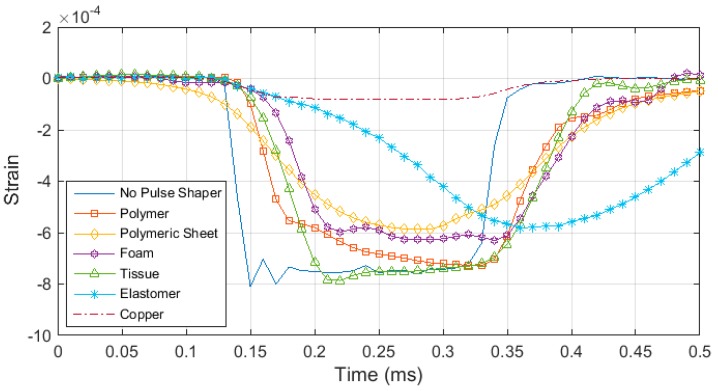
A comparison of pulse profiles generated by different kind of material as the pulse shaper.

This section presents the analysis made on pulse shapers which are placed between the striker and incident bar to (i) reduce the wave dispersion by physically filtering out the high frequency components in the generated pulse through elastic–plastic deformation and (ii) to facilitate stress equilibrium by increasing the rise time of the loading pulse. By using a pulse shaper, various profiles of loading pulses can be generated to characterize materials like elastomers, having characteristics that are affected by loading shape of the pulse. The profile of incident wave generated through the pulse shaping technique is dependent on the geometry and material of the pulse shaper as well as the impact velocity of the striker. Such an example is shown in Figure 8, in which experimental results for different profiles of incident pulses are illustrated by utilizing various different materials widely used for this purpose.

**Figure 9:**
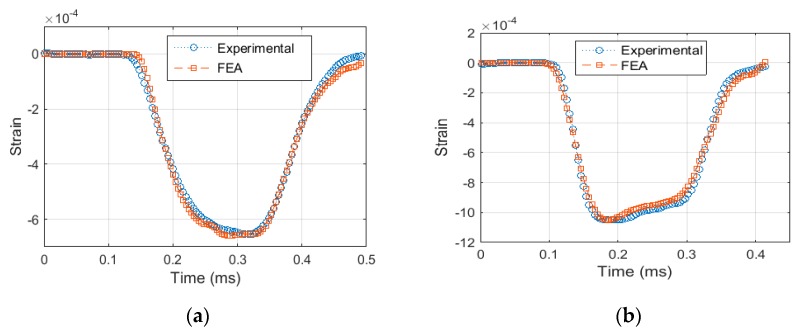
Experimental and FEM results for the incident pulse generated by a copper pulse shaper under different impact velocities. (**a**) 6.6 m/s (**b**) 11.0 m/s.

Copper is the most popular choice for the pulse shaping technique, as it is able to generate a loading pulse with an extended rise time and a constant state of stress for the most part of the loading. In the previous sections, we described the methodology to model the behavior of an annealed copper disc (CU11000) when used as the pulse shaper. Figure 9 shows the experimental results and the FEM validation of the pulses generated by using a copper disc (thickness of 1.5 mm and diameter of 20 mm) when impacted at different velocities. It is worth noting that the generate pulses are different not only in amplitude but as well as the shape due to the strain rate dependent plastic deformation in copper.

**Figure 10:**
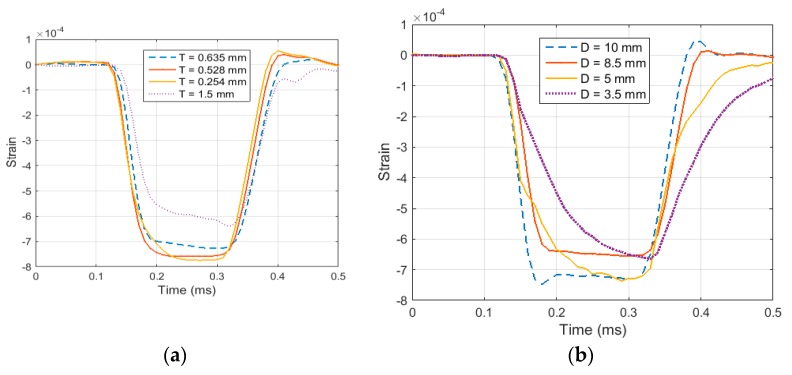

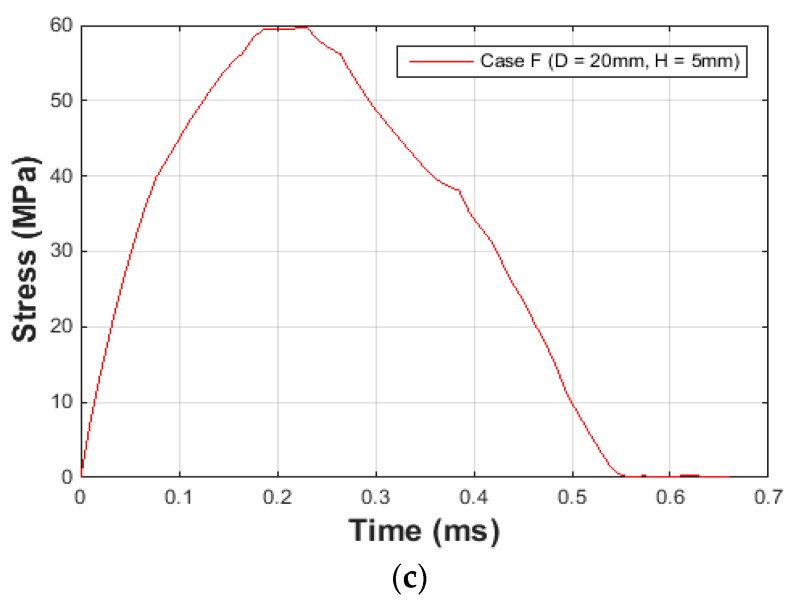
(**a**) Incident pulses generated from pulse shapers of various thickness with a striking velocity of 7.5 ms^−1^ (**b**) Incident pulses generated from pulse shapers of various diameters with a striking velocity of 7.5 ms^−1^. (**c**) Hybrid copper and elastomeric pulse shaper.

Another challenge is to determine the optimal geometry of pulse shaper and analyze the effect of the varying geometry size on the generated pulse. For this purpose, different geometry sizes were analyzed experimentally. First, the diameter was kept constant and the model was run with varying thicknesses of the pulse shaper. Figure 10a shows the resultant incident pulse for a copper pulse shaper with various thicknesses and diameter of 20 mm. Then, the thickness was kept constant at 0.635 mm, and different cases were analyzed by varying the diameter (see Figure 10b). It can be noted from Figure 10a that increasing the pulse shaper thickness results in a longer rise time and an increased pulse duration. Moreover, the pulse shaper thickness also effects the region of plastic deformation that is evident when the thickness is increased from 0.635 mm to 1.5 mm. Similarly, Figure 10b shows that decreasing the diameter results in an increased rise time and a longer duration of incident pulse with an early start region of plastic deformation. Figure 10c illustrates the FEA results for the stress history of the incident pulse generated by employing a hybrid elastomer/copper pulse shaper. The resultant pulse exhibits an extended and similar rise time and fall time reaching a constant stress amplitude.

The experimental and numerical results show that the incident pulse shape depends upon the material, geometry of the pulse shaper and the striking velocity of the striker. By optimizing these parameters, an incident pulse suitable to test elastomers can be generated in a Kolsky bar technique. The criteria for an acceptable pulse shaper are homogenous stress distribution and a constant rate of deformation. Any pulse shaper design is acceptable as long as it fulfills this criterion.

### 4.3. High Strain Rate Response of NBR

**Figure 11:**
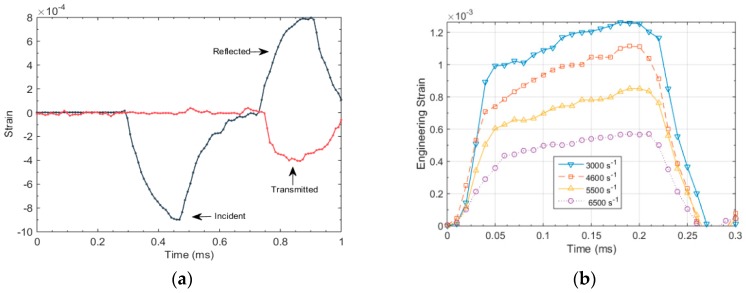
(**a**) Typical incident, reflected and transmitted pulses in a Kolsky bar experiment; (**b**) Incident pulses at different strain rates.

In this section, we present Kolsky compression bar experiments performed on nitrile butadiene (NBR) rubber. The material came in the form of sheets 2.4 mm thick from which cylindrical specimen of diameter 8.5 were cut. Figure 11a shows an incident, reflected and transmitted pulses in a Kolsky bar experiment performed at a strain rate of 3000 s^−1^. An annealed copper disk (CU11000) was employed as the pulse shaper to characterize NBR specimens. The dimensions of the pulse shaper were selected to be 8.5 mm in diameter and 0.635 mm in thickness. This resulted in an incident pulse with enough of an extended rise time for the specimen to attain stress equilibrium and a constant strain rate of deformation for the most part of loading. Figure 11b illustrates the incident pulses generated at different strain rates ranging from 3000 s^−1^ to 6500 s^−1^.

**Figure 12:**
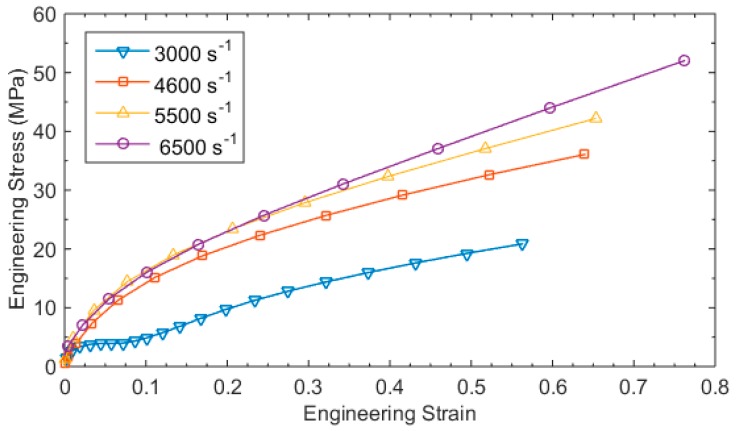
Compressive stress strain curves for NBR at different strain rates.

The incident and reflected pulse were used to compute the strain history in the test specimen using Equation (16), and the stress history was computed using Equation (7). Figure 12 illustrates the engineering strain vs stress curves for different dynamic strain rates varying between 3000 s^−1^ and 6500 s^−1^. The response of the test specimen showed significant strain rate effects while exhibiting similar trend in curves. The typical characteristics exhibited in the test sample includes a near linear behavior at small strains followed by a nonlinear transition towards strain hardening region and lastly the unloading of the specimen which is typical in rubber samples. During the unloading of the specimen, the viscoelastic characteristics of elastomers assists recovery of the sample with a very small stress amplitude.

## 5. Conclusions

For the characterization of elastomers at strain rates of 100 s^−1^–10,000 s^−1^, a modified Kolsky bar was developed. In the design of such an apparatus, important considerations such as homogenous deformation of the test specimen, hollow transmission rod, and pulse shaping techniques were taken into account. A complete methodology to numerically model Kolsky bar in FEA is also described, which was used to optimize the specimen geometry and to study the effect of homogenous deformation considering various dimensions for the specimen. Experimental and numerical analysis was made on the pulse shaping technique to analyze the effect of thickness and diameter of the pulse shaper on the incident pulse. Moreover, resultant incident pulses generated from commonly used materials for pulse shapers were also taken into account and a design for a hybrid elastomer/copper pulse shaper is also discussed. Finally, employing the optimized geometry of the specimen and the pulse shaper, response of NBR rubber is investigated at various different strain rates.

## Figures and Tables

**Table 1:** Johnson-Cook and Equation of State input parameters for pulse shaper and Ogden parameters for specimen.

Model	Parameters					
J-C	A	B	C	n	m	${\dot{\varepsilon }}_{0}$
	92 × 10^6^	292 × 10^6^	0.025	0.31	1.09	1
U_s-_U_p_	c_o_	S		$\Gamma_{0}$		
	3933	1.5		1.99		
Ogden	$\mu_{i}$	$\alpha^{i}$		$D_{i}$		
	$\mu_{1}$: 365,227.2	$\alpha^{1}$: 1.544		$D_{1}$: 1.00 × 10^−8^		
	$\mu_{2}$: 643.74	$\alpha^{2}$: 5.846		$D_{2}$: −1.41 × 10^−8^		
	$\mu_{3}$: 16,712.2	$\alpha^{3}$: −1.834		$D_{3}$: 4.87 × 10^−10^		
